# Supplementary material for: Decorated Electrode Surfaces with Nanostructures and Metal-Organic Frameworks as Transducers for Sensing
Source: Sensors (Basel). 2024 Oct 20;24(20):6745. doi: 10.3390/s24206745 (PMC11511523; doi:10.3390/s24206745)
Supplement: Supplementary file 1 [file sensors-24-06745-s001.zip › sensors-3155795-supplementary.pdf]

Supplementary information

Decorated electrode surfaces with nanostructures and metal-organic frameworks as transducers for sensing

Sara Caruncho-Pérez[a,b,*], Aida M. Díez[b], Ana Prado-Comesaña[a,b], Marta Pazos[b], María Ángeles Sanromán[b], Elisa González-Romero [a,*]

[a]Department of Analytical and Food Chemistry, University of Vigo, Campus As Lagoas-Marcosende, 36310 Vigo, Spain; ana.maria.prado.comesana@uvigo.es

[b]Campus As Lagoas-Marcosende, CINTECX—University of Vigo, 36310 Vigo, Spain; adiez@uvigo.es (A.M.D.); mcurras@uvigo.es (M.P.); sanroman@uvigo.es (M.Á.S.)

*Correspondence: sara.caruncho@uvigo.es (S.C.-P.); eromero@uvigo.es (E.G.-R.)

Table S1. Data extracted from the voltammograms for $[Fe(CN)_6]^{3-}$ with SPCEs modified with nanomaterials.

SPCE	$i_{p,a}$ (μA)	$E_{p,a}$ (V)	$-i_{p,c}$ (μA)	$E_{p,c}$ (V)	$\vert i_{p,a}/i_{p,c}\vert$	$E_{p,a}$ - $E_{p,c}$ (V)
Unmodified	52.17	0.241	42.00	-0.121	1.24	0.362
GPH	47.97	0.201	56.56	-0.011	0.85	0.212
MWCNT	62.52	0.186	71.78	-0.016	0.91	0.202
AuNP	49.77	0.271	49.27	-0.051	1.01	0.322
CNT-AuNP	68.53	0.155	73.23	-0.016	0.94	0.171
FeNP	27.15	0.377	14.14	-0.242	1.92	0.619
CNF	49.86	0.216	54.03	-0.036	0.92	0.252

Table S2. Data extracted from the main peaks of CLT and SMX with SPCEs modified with nanomaterials.

Analyte	SPCE	i_p (μA)	E_p (V)	$W_{1/2}$ (V)	i_p improvement (%)
	Unmodified	6.506	0.774	0.098	-
	GPH	7.766	0.678	0.073	19
SMX	MWCNT	5.983	0.734	0.160	-8
	AuNP	6.774	0.759	0.124	4
	CNT-AuNP	6.109	0.729	0.154	-6

	FeNP	4.677	0.794	0.094	-28
	CNF	6.518	0.764	0.119	0
CLT	Unmodified	11.291	0.827	0.093	-
	GPH	17.649	0.792	0.093	56
	MWCNT	14.864	0.812	0.094	32
	AuNP	11.686	0.822	0.086	3
	CNT-AuNP	17.450	0.817	0.091	54
	FeNP	10.331	0.857	0.085	-8
	CNF	11.470	0.817	0.097	2

Table S3. Data extracted from the Nyquist plot for 5 mM $[Fe(CN)_6]^{3-}$ in 10 mM Na_2SO_4.

SPCE	R_s (Ω)	R_{ct} (Ω)	α
Unmodified	245	2600	0.98
MWCNT	184	2830	0.40
CNT-AuNP	181	2120	0.45
MOF Ti	259	3640	0.84
MOF Fe	262	1680	0.68
AQ	290	84500	0.95
CA	255	8940	0.95
Nafion	262	110000	0.56
Chitosan	280	2090	0.72

Table S4. Data extracted from the voltammograms for $[Fe(CN)_6]^{3-}$ with SPCEs modified with the MOFs.

SPCE	$i_{p,a}$ (μA)	$E_{p,a}$ (V)	$-i_{p,c}$ (μA)	$E_{p,c}$ (V)	$\lvert i_{p,a}/i_{p,c}\rvert$	$E_{p,a} - E_{p,c}$ (V)
Unmodified	47.15	0.231	34.82	-0.056	1.35	0.287
MOF Ti	61.82	0.211	55.79	-0.066	1.11	0.277
MOF Fe	73.96	0.186	80.54	-0.016	0.92	0.202

Table S5. Data extracted from the voltammograms for $[Fe(CN)_6]^{3-}$ with SPCEs modified with the Ti-based MOF and the corresponding polymers.

SPCE	$i_{p,a}$ (μA)	$E_{p,a}$ (V)	$-i_{p,c}$ (μA)	$E_{p,c}$ (V)	$\lvert i_{p,a}/i_{p,c}\rvert$	$E_{p,a} - E_{p,c}$ (V)
Unmodified	52.89	0.221	41.36	-0.066	1.28	0.287
MOF Ti	63.38	0.206	55.10	-0.076	1.15	0.282
AQ	4.41	0.342	2.50	-0.318	1.76	0.660
MOF/AQ	1.41	0.432	3.18	-0.172	0.44	0.604
AQ/MOF	0.53	0.458	0.86	0.040	0.62	0.418

CA	11.41	0.160	9.38	-0.006	1.22	0.166
MOF/CA	6.49	0.342	4.78	-0.308	1.36	0.650
CA/MOF	12.72	0.241	10.40	-0.142	1.22	0.383
Nafion	4.59	0.407	0.17	2.674	1.72	0.237
MOF/Nafion	-	-	-	-	-	-
Nafion/MOF	-	-	-	-	-	-
Chitosan	144.62	0.170	135.4	0.014	1.07	0.156
MOF/Chitosan	150.91	0.206	137.0	0.004	1.10	0.202
Chitosan/MOF	143.02	0.201	159.1	-0.031	0.90	0.232

Table S6. Data extracted from the voltammograms for $[Fe(CN)_6]^{3-}$ via electropolymerization and diazotization.

**SPCE**	**$i_{p,a}$ (μA)**	**$E_{p,a}$ (V)**	**$-i_{p,c}$ (μA)**	**$E_{p,c}$ (V)**	**$\|i_{p,a}/i_{p,c}\|$**	**$E_{p,a}$ - $E_{p,c}$ (V)**
Unmodified	66.18	0.201	58.85	-0.061	1.12	0.262
MOF Ti	68.19	0.181	65.90	-0.046	1.03	0.227
MOF Ti/po-PD	36.39	0.140	52.47	-0.026	0.70	0.166
po-PD/MOF Ti	34.65	0.145	38.21	-0.071	0.91	0.216
MOF Fe	79.68	0.170	80.21	-0.006	0.99	0.176
MOF Fe/po-PD	60.53	0.140	77.71	-0.026	0.78	0.166
po-PD/MOF Fe	38.39	0.125	48.17	-0.031	0.80	0.156
Diazotization ATA	Missing signal	Missing signal	Missing signal	Missing signal	-	-
Diazotization MOF Ti	Missing signal	Missing signal	Missing signal	Missing signal	-	-
Diazotization MOF Fe	Missing signal	Missing signal	Missing signal	Missing signal	-	-

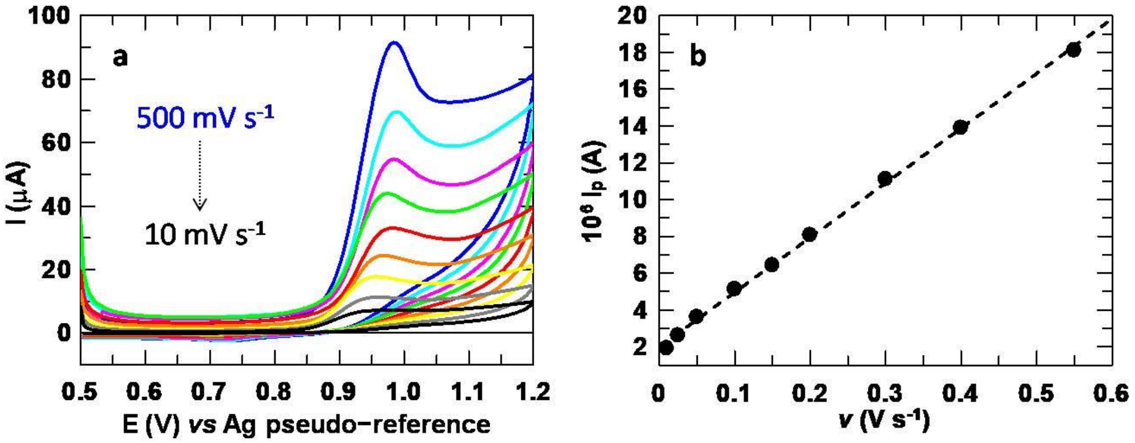

Figure S1. (a) Voltammograms obtained by CV during the scan rate study of CLT 151.2 µM in Na_2SO_4 10 mM at pH 3. (b) Fitted curve obtained after plotting the peak intensity of CLT *vs* the scan rate.

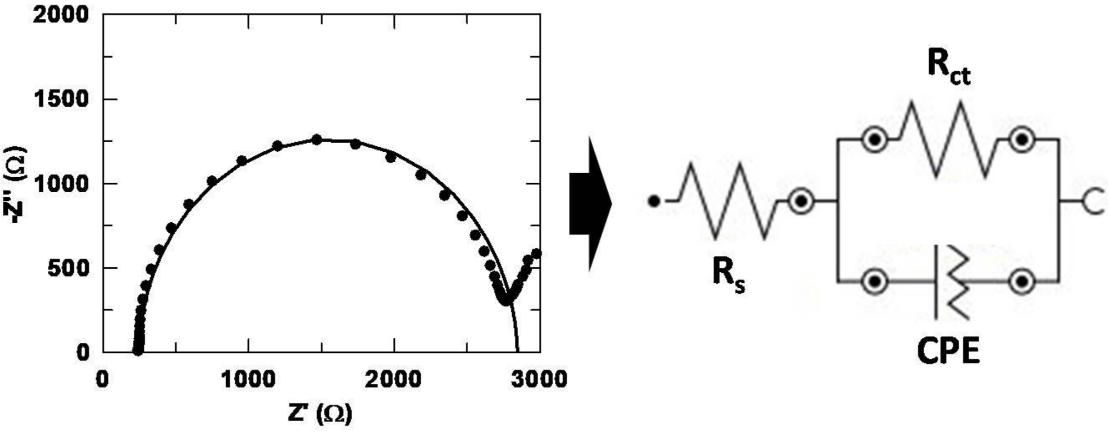

Figure S2. Nyquist plot of the unmodified SPCE with the corresponding fit derived from the equivalent circuit shown on the right.

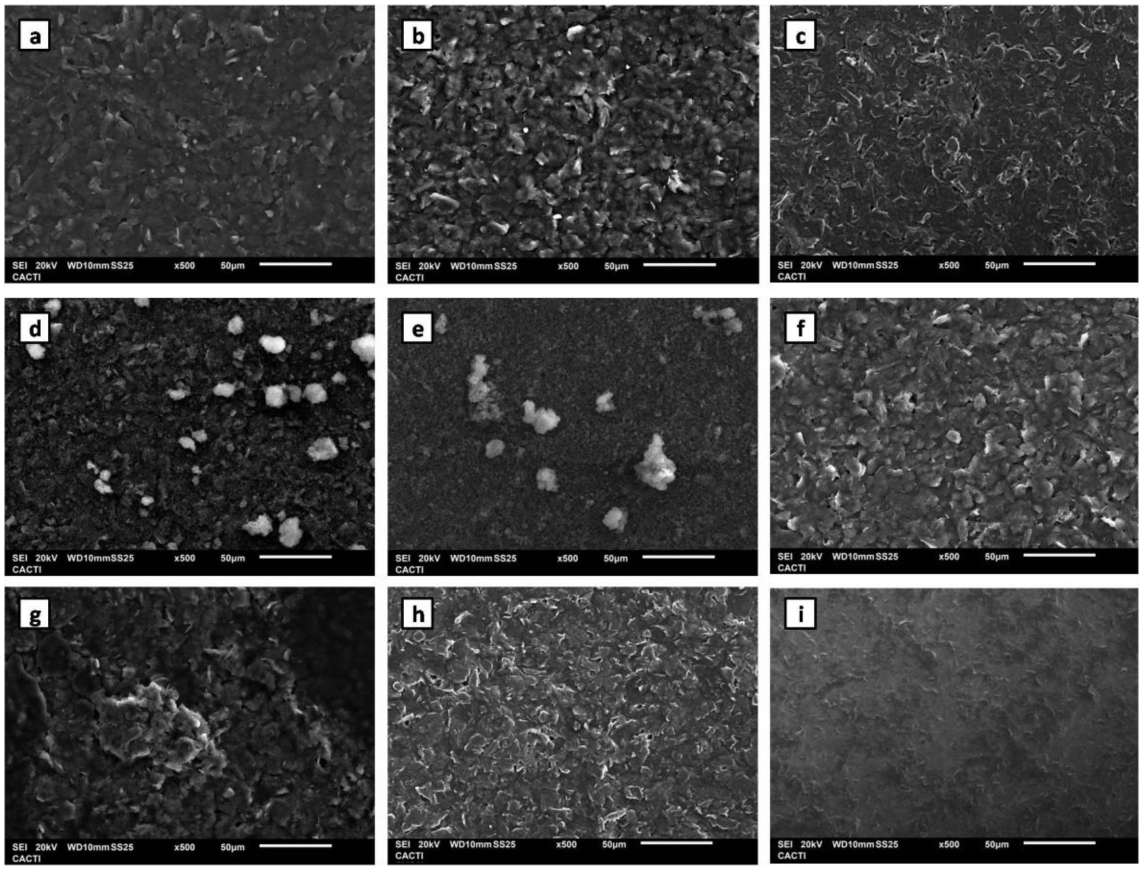

Figure S3. SEM images with a magnification ×500 of (a) unmodified SPCE, (b) SPCE/MWCNT, (c) SPCE/CNT-AuNP, (d) SPCE/MOF Ti, (e) SPCE/MOF Fe, (f) SPCE/AQ, (g) SPCE/CA, (h) SPCE/Nafion and (i) SPCE/Chitosan.

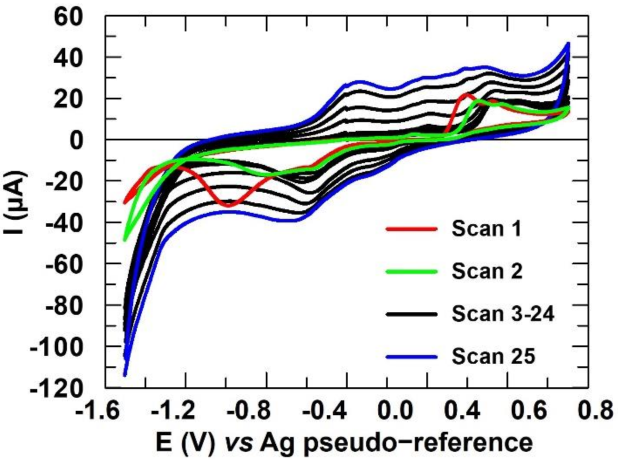

Figure S4. Voltammograms obtained by CV during the electropolymerization of o-PD.

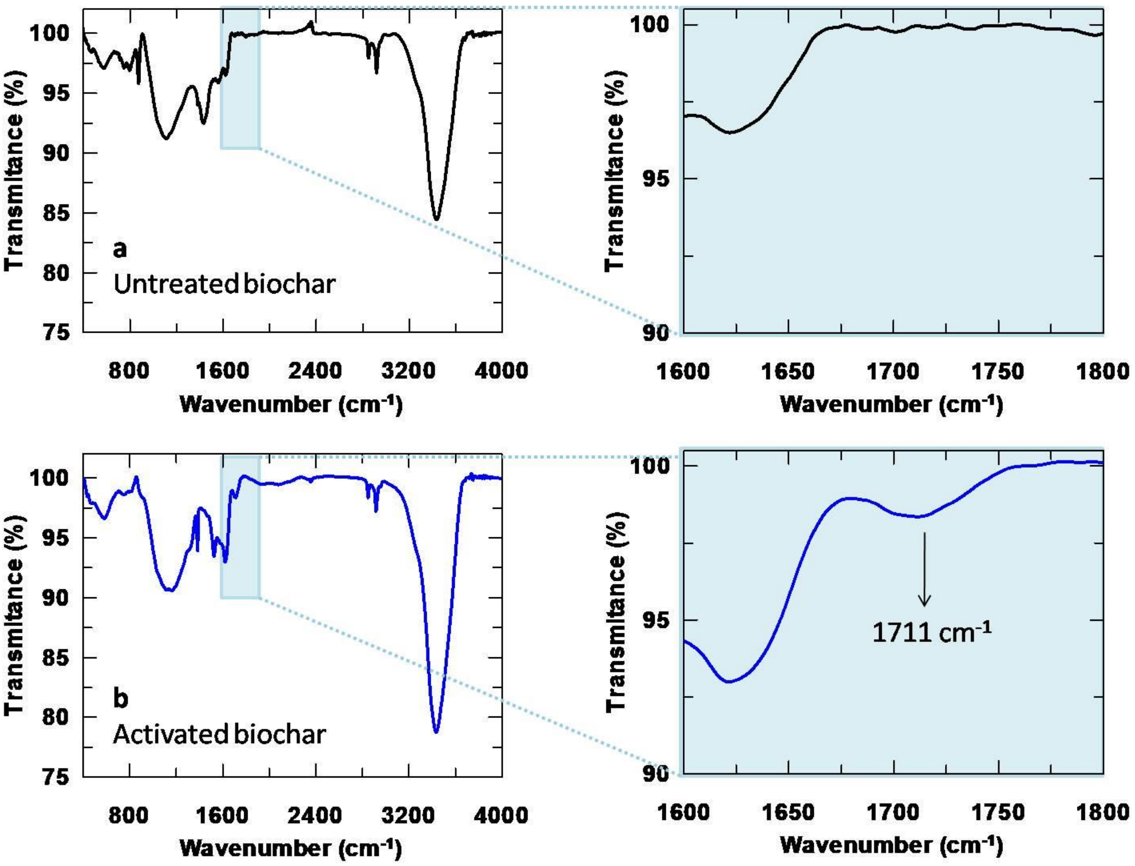

Figure S5. FTIR spectra of (a) untreated and (b) activated biochar with their corresponding amplification between 1600 and 1800 cm^{-1}.

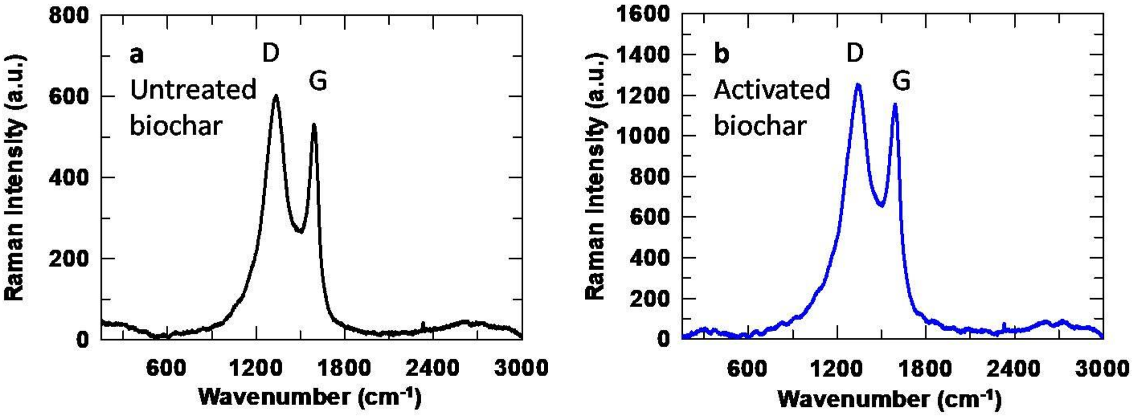

Figure S6. Raman spectra of (a) untreated and (b) activated biochar.
